# To Contrast or Not to Contrast? On the Role of Contrast Enhancement in Hand MRI Studies of Patients with Rheumatoid Arthritis

**DOI:** 10.3390/diagnostics12020465

**Published:** 2022-02-11

**Authors:** Miriam Frenken, Gesa Rübsam, Alexander Mewes, Karl Ludger Radke, Lien Li, Lena M. Wilms, Sven Nebelung, Daniel B. Abrar, Philipp Sewerin

**Affiliations:** 1 Institute of Diagnostic and Interventional Radiology, University Hospital of Düsseldorf, Moorenstraße 5, 40225 Dusseldorf, Germany; alexanderdieter.mewes@med.uni-duesseldorf.de (A.M.); ludger.radke@med.uni-duesseldorf.de (K.L.R.); lena.wilms@med.uni-duesseldorf.de (L.M.W.); sven.nebelung@med.uni-duesseldorf.de (S.N.); danielbenjamin.abrar@med.uni-duesseldorf.de (D.B.A.); 2 Department and Hiller Research Unit of Rheumatology, Heinrich Heine University Düsseldorf, UKD, Moorenstrasse 5, 40225 Düsseldorf, Germany; gesa.ruebsam@med.uni-duesseldorf.de (G.R.); philipp.sewerin@med.uni-duesseldorf.de (P.S.); 3 Institute for Medical Information Processing, Biometry and Epidemiology, Ludwig-Maximilians-Universität München, Marchioninistr. 15, 81377 Munich, Germany; le-lien@ibe.med.uni-muenchen.de; 4 Department of Diagnostic and Interventional Radiology, Aachen University Hospital, 52074 Aachen, Germany; 5 Rheumazentrum Ruhrgebiet Herne, Ruhr-University Bochum, 44649 Herne, Germany

**Keywords:** MRI, gadolinium, gadolinium-based MRI contrast agent, rheumatoid arthritis, RAMRIS, synovitis, musculoskeletal imaging

## Abstract

Currently, clinical indications for the application of gadolinium-based contrast agents (GBCA) in magnetic resonance imaging (MRI) are increasingly being questioned. Consequently, this study aimed to evaluate the additional diagnostic value of contrast enhancement in MRI of the hand in patients with rheumatoid arthritis (RA). Thirty-one patients with RA (mean age, 50 ± 14 years (range, 18–72 years)) underwent morphologic MRI scans on a clinical 3 T scanner. MRI studies were analyzed based on (1) the Rheumatoid Arthritis Magnetic Resonance Imaging Score (RAMRIS) and (2) the GBCA-free RAMRIS version, termed RAMRIS Sine-Gadolinium-For-Experts (RAMRIS-SAFE), in which synovitis and tenosynovitis were assessed using the short-tau inversion-recovery sequence instead of the post-contrast T1-weighted sequence. The synovitis subscores in terms of Spearman’s *ρ*, as based on RAMRIS and RAMRIS-SAFE, were almost perfect (*ρ* = 0.937; *p* < 0.001), while the tenosynovitis subscores were less strongly correlated (*ρ* = 0.380 *p* = 0.035). Correlation between the total RAMRIS and RAMRIS-SAFE was also almost perfect (*ρ* = 0.976; *p* < 0.001). Inter-rater reliability in terms of Cohen’s *κ* was high (0.963 ≤ *κ* ≤ 0.925). In conclusion, RAMRIS-SAFE as the GBCA-free version of the well-established RAMRIS is a patient-friendly and resource-efficient alternative for assessing disease-related joint changes in RA. As patients with RA are subject to repetitive GBCA applications, non-contrast imaging protocols should be considered.

## 1. Introduction

Rheumatoid arthritis (RA) is a chronic autoimmune disease affecting small joints of the hand and the feet, characterized by pain, inflammation, and joint destruction that can lead to functional disability and severe reduction in quality of life, followed by a significant economic burden for individual patients and society as a whole [1,2,3]. A variety of different therapies are available for RA. A treat-to-target approach is advised by the American College of Rheumatology [4], with regular assessments and treatment adjustment if the response is inadequate [4]. To ensure comprehensive monitoring under therapy, sensitive diagnostic tools are beneficial [5,6,7].

Magnetic resonance imaging (MRI) provides insight into disease status and treatment response by accurate assessment of inflammation and structural damage in joints [8]. RA-typical findings can be assessed by the semiquantitative outcome measures in rheumatology (OMERACT) RA-MRI scoring system (RAMRIS), a sum score that evaluates inflammatory (i.e., osteitis/bone marrow edema, synovitis, tenosynovitis) and destructive changes (i.e., erosions, cartilage loss/joint space narrowing (JSN)) in the metacarpophalangeal (MCP) joints, hand, and wrist [8,9].

Intravenous administration of gadolinium-based contrast agents (GBCA) is currently required to perform MRI studies with RAMRIS assessment in accordance with OMERACT recommendations, as the use of the T1-weighted post-contrast sequences is still recommended, particularly for assessment of synovitis [10]. These sequences are similarly recommended for the evaluation of tenosynovitis, although unenhanced water-sensitive sequences may be considered as alternatives [9].

Lately, gadolinium deposition in the brain has been reported after repetitive administration of linear GBCAs [11,12,13,14]. Consequently, the Food and Drug Administration (FDA) reviewed GBCAs and concluded that although gadolinium retention was not directly associated with adverse effects in patients with normal renal function, a new class warning was required [15]. The European Medicines Agency has also responded with a recommendation to discontinue the use of these GBCAs [15]. More stable cyclic GBCAs may still be applied but should only be used when unenhanced studies are insufficient [16]. Thus, with regards to the growing safety concerns among clinicians and regulators, there has been a strong push towards GBCA-free MRI study protocols.

The objective of this study was to comparatively evaluate the diagnostic performance of a GBCA-free MRI protocol of the hand called “RAMRIS-Sine-Gadolinium-For-Experts (RAMRIS-SAFE)” versus the standard contrast-enhanced MRI protocol (RAMRIS) when evaluating disease activity in patients with RA. Our hypotheses were that (a) RAMRIS-SAFE is diagnostically non-inferior to RAMRIS, both in terms of the sum score as well as the subscores for synovitis and tenosynovitis, and that (b) the Short-Tau Inversion-Recovery (STIR) sequence is an appropriate alternative for the T1-weighted postcontrast sequence.

## 2. Materials and Methods

### 2.1. Study Design and Patients

In this prospective intra-individual comparison study, 31 adult patients (mean age 50 ± 14 years, (range 18–72 years), 18 females) with active RA based on the American College of Rheumatology criteria [17] (i.e., arthritis ≥ 6 weeks, at least two affected joints or morning stiffness > 30 min and at least one affected joint) were included. High-resolution MRI studies of the clinically more affected hand were obtained using a dedicated 16-channel high-resolution hand coil (3 T Tim Coil, Siemens Healthineers, Erlangen, Germany) and a clinical 3 T MRI scanner (3 T MAGNETOM Skyra, Siemens Healthineers, Erlangen, Germany). Clinical disease activity in terms of blood markers of inflammation (i.e., C-reactive protein (CRP)) and the Disease Activity Score 28 (DAS-28) using CRP for its calculation, were obtained, as well, when patients were recruited. DAS-28 was determined by the senior author (PS, clinical rheumatologist with 12 years of experience), at recruitment, for each patient. In this study, DAS-28 levels were categorized, according to previously validated cutoff values [18], into remission or low disease activity (0–3.2), moderate disease activity (>3.2–5.1), and high disease activity (>5.1). CRP levels had been determined during routine laboratory assessment by the hospital’s central laboratory and were retrieved from the patients’ medical records (unit: mg/dL). The reference range was <0.5 mg/dL (normal).

The study was conducted in line with the Declaration of Helsinki and approved by the local ethics committee (protocol code 3828). Written informed consent was obtained from all patients.

### 2.2. MRI Acquisition

**Figure 1:**
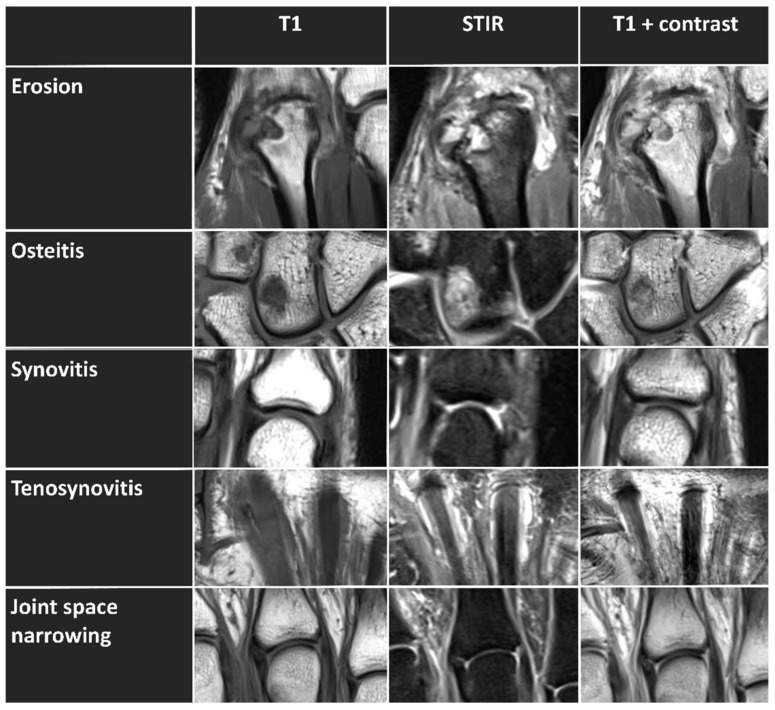
Exemplary imaging features (erosion, osteitis, synovitis, tenosynovitis, and joint space narrowing) that are scored semiquantitatively based on RAMRIS, shown in unenhanced MRT T1-weighted sequence (left column), STIR sequence (middle column), and enhanced T1-weighted postcontrast sequence (right column). Erosion is visualized at the bare area of the distal metacarpal bone of the second digit (i.e., the MCP joint base) of the right hand of a 48-year-old male patient. Osteitis is visualized in the right carpal bones with bone marrow edema in the capitate and trapezoid of a 56-year-old female patient. Synovitis is visualized in the right metacarpophalangeal joint of the fifth digit of a 53-year-old female patient. Tenosynovitis is visualized around the right flexor tendon of the second and third digit of a 75-year-old female patient. In the last row, metacarpophalangeal joints of the right hand with normal joint space without evidence of cartilage loss of a 52-year-old female patient are shown.

Unilateral MRI studies of the wrist, hand, and MCP joints were performed in the prone position with the hand above the head and the palm facing down (superman position). The clinically more affected hand (*n* = 25 (right hand), *n* = 6 (left hand)) was selected to assess the following inflammatory and destructive changes: erosion, osteitis (bone marrow edema), synovitis, tenosynovitis, and JSN (as a sign of cartilage loss). Erosions, osteitis, and JSN were assessed using axial and coronal T1-weighted turbo spin-echo (TSE) and coronal STIR sequences without exogenous contrast enhancement (Figure 1). Semiquantitative assessment of synovitis and tenosynovitis based on RAMRIS was performed using contrast-enhanced coronal T1-weighted TSE sequences without fat suppression and axial T1-weighted spin-echo (SE) sequences with spectral fat saturation (in the following, referred to as “synovitis-contrast” and “tenosynovitis-contrast”). To this end, gadoterate meglumine (Gd-DOTA, Dotarem, Guerbet, Roissy CdG CEDEX, France) was injected intravenously, and a delay of 6 min was chosen until the post-contrast sequences were acquired.

Correspondingly, semiquantitative assessment of synovitis and tenosynovitis based on RAMRIS-SAFE was performed using coronal STIR and axial T1-weighted TSE sequences (referred to as “synovitis-SAFE” and “tenosynovitis-SAFE”). Table 1 details which sequences were used to assess the individual imaging features. The detailed MRI protocols are listed in Table 2.

### 2.3. MRI Evaluation

MR images were analyzed in consensus by two clinical radiologists with special expertise in musculoskeletal imaging (A.M., 12 years; M.F., 6 years) who were blinded to the patient data and semiquantitatively evaluated erosion, osteitis, and JSN. Since the presence of contrast enhancement was easily discernible, blinding proved impossible during scoring of synovitis and tenosynovitis.

### 2.4. Statistical Analysis

Statistical analysis was performed by L.R. and M.F. using the statistical software SPSS (v28, SPSS Inc., Chicago, IL, USA). Based on both radiologists’ subscores (i.e., erosion, osteitis, synovitis-contrast, synovitis-SAFE, tenosynovitis-contrast, tenosynovitis-SAFE, and JSN), sum scores were calculated for RAMRIS and RAMRIS-SAFE. For correlation analysis of these sum scores and subscores, as well as for DAS-28 and CRP levels, Spearman’s rank correlation coefficients ρ were calculated. The effect size ρ was categorized as small (0.1–0.3), medium (0.3–0.5), and strong (>0.5), according to Cohen et al. [19]. For assessing inter-rater reliability, Cohen’s *κ* was calculated and classified according Landis and Koch as poor (*κ* ≤ 0), slight (0 < *κ* ≤ 0.2), fair (0.2 < *κ* ≤ 0.4), moderate (0.4 < *κ* ≤ 0.6), substantial (0.6 < *κ* ≤ 0.8), and almost perfect (*κ* > 0.8) [20]. Inter-rater reliability was quantified by Cohen’s *κ*. Due to the study’s exploratory nature, *p*-values ≤ 0.05 were considered significant. 

## 3. Results

### 3.1. Comparison of Sum Scores and Subscores

**Figure 2:**
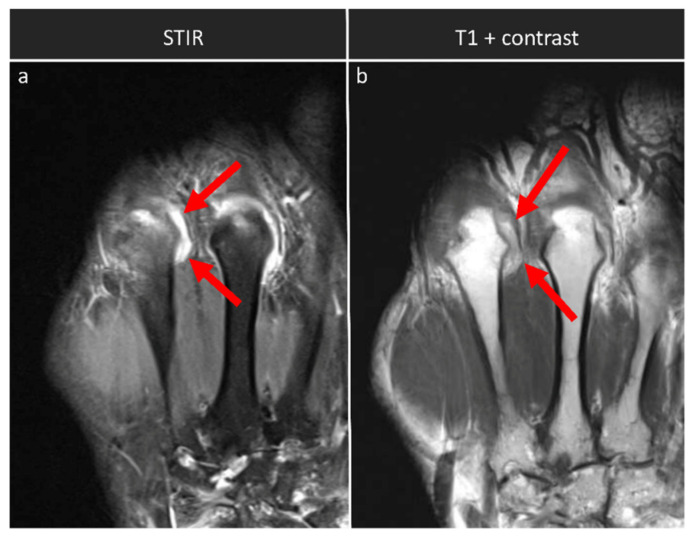
Synovitis in an exemplary patient across the sequences. This coronal section of a STIR sequence (**a**) and a T1-weighted post-contrast sequence without fat suppression (**b**) visualizes synovitis at joint effusion at the ulnar aspect of the MCP II joint of the right hand of a 51-year-old female patient. Synovial thickening and effusion as signs of synovitis are visible in both sequences (red arrows in (**a**,**b**)).

**Figure 3:**
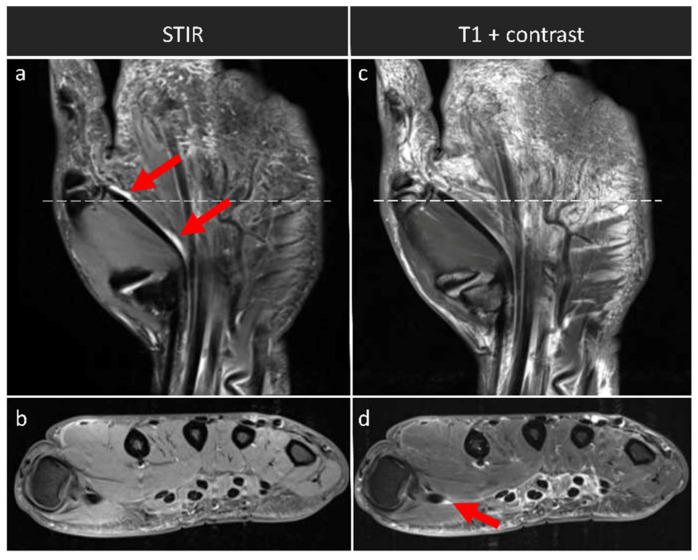
Tenosynovitis in an exemplary patient across the sequences. Coronal STIR (**a**) and axial T1-weighted sequence with fat suppression, yet without contrast agent application (**b**) and corresponding coronal (**c**) and axial (**d**) T1-weighted post-contrast sequences. Axial images were acquired at the level of distal metacarpal bones (as indicated by the white horizontal lines). Tenosynovitis of the flexor tendon of the first digit can be identified in the STIR sequence (dual red arrows in (**a**) and in the contrast-enhanced sequences (single red arrow in (**d**)). Right hand of a 48-year-old male patient.

We found a strong and significant correlation for the synovitis subscores, i.e., synovitis-SAFE and synovitis-contrast (*ρ* = 0.937; *p* < 0.001). Qualitative comparative evaluation of the STIR and the T1-weighted post-contrast sequences indicated corresponding imaging findings (Figure 2). For the assessment of tenosynovitis, the two subscores, i.e., tenosynovitis-SAFE and tenosynovitis-contrast, correlated only on a medium level, yet still significantly (*ρ* = 0.380; *p* = 0.035). Qualitatively, tenosynovitis was visible in the STIR and post-contrast T1-weighted sequences (Figure 3). Between RAMRIS and RAMRIS-SAFE, there was a strong and significant correlation (*ρ* = 0.976; *p* < 0.001).

The subscores of erosion, osteitis, JSN, and synovitis were found to display variable yet significant pair-wise correlations among each other. In contrast, tenosynovitis, i.e., tenosynovitis-contrast and tenosynovitis-SAFE, was only significantly correlated with the corresponding synovitis subscore, i.e., synovitis-SAFE (tenosynovitis-SAFE vs. synovitis-SAFE: *ρ* = 0.381; *p* = 0.034) and tenosynovitis-contrast (tenosynovitis-contrast vs. synovitis-contrast: *ρ* = 0.579; *p* < 0.001). DAS-28 correlated significantly with all RAMRIS and RAMRIS-SAFE sum scores and subscores, except for both tenosynovitis scores. CRP levels correlated significantly only with DAS-28 but not with any MRI score, as shown in Table 3.

Statistical variation of synovitis and tenosynovitis was approximately similar, regardless of the presence or absence of contrast enhancement, which indicates the absence of systematic bias, i.e., overestimation or underestimation, secondary to contrast enhancement (Table 3). Furthermore, results were as follows: RAMRIS mean 33.55 ± 34.94 (range, 0–129), RAMRIS-SAFE mean 33.26 ± 34.36 (range, 0–129), osteitis mean 4.06 ± 7.77 (range, 0–32), erosion mean 10.39 ± 12.95 (range, 0–42), and JSN mean 8.03 ± 12.47 (range, 0–47).

DAS-28 levels ranged from 0.1 to 6.6 with a mean of 4.13 ± 1.41. The number of patients per category was *n* = 13 (remission or low disease activity), *n* = 7 (moderate disease activity), and *n* = 11 (high disease activity). CRP values ranged from 0.1 mg/dL to 9.4 mg/dL with a mean of 1.55 ± 2.15 mg/dL.

### 3.2. Interrater Reliability

Cohen’s kappa analysis revealed almost perfect interrater agreement for the RAMRIS subscores synovitis-contrast (*κ* = 0.950; *p* < 0.001) and tenosynovitis (*κ* = 0.925; *p* < 0.001) as well as for RAMRIS-SAFE subscores synovitis-contrast (*κ* = 0.963; *p* < 0.001) and tenosynovitis (*κ* = 0.929; *p* < 0.001).

## 4. Discussion

The most important finding of this study is that exogeneous contrast enhancement is not necessary to reliably score synovitis and tenosynovitis within the framework of RAMRIS. Consequently, patients undergoing MRI of the hand to determine disease activity in RA can be assessed accurately using the GBCA-free version of RAMRIS, i.e., RAMRIS-SAFE, without compromising diagnostic performance.

Exogeneous contrast enhancement appears not to be relevant for the imaging-based scoring of bone erosion and bone edema in the wrist and MCP joints in patients with RA [10], and, presumably, the same applies to JSN. Consequently, the modified RAMRIS-SAFE differed from the original RAMRIS only in the subscores for synovitis and tenosynovitis. Both features were assessed based on the STIR sequence instead of the T1-weighted post-contrast sequence. There was a strong correlation between the unenhanced and enhanced synovitis subscores, which suggests that synovitis can be well detected and graded using the fluid-sensitive STIR sequence. These results contrast with previous findings by Østergaard et al., which led to the OMERACT recommendation to use GBCA for the evaluation of synovitis in RAMRIS [9,10]. Unlike the previous study that used substantially lower field strengths of 0.2 T, 1 T, and 1.5 T, our study was conducted using a stronger field strength of 3 T, a dedicated multi-channel hand coil, and sequences that are optimized in terms of image resolution. Consequently, we realized imaging at relatively high in-plane image resolution of 0.31 × 0.45 mm/pixel at a slice thickness of 2.5 mm. This contrasts with the image settings of Østergaard et al., where the STIR sequence was acquired at 0.73 × 0.88 mm/pixel (0.2T) and 0.59 × 0.57 mm/pixel (1 T), respectively, with a slice thickness of 3 mm, and a T2-weighted sequence was acquired at 0.51 × 0.39 mm/pixel (1.5 T) with slice thickness of 2 mm; this may explain the discrepancy outlined above. Moreover, image resolution needs to be balanced against acquisition time and, at 4:23 min, our STIR sequence is suitable for clinical workflow and is in use. Another factor to consider is the use of the 16-channel high-resolution hand coil, which is closely centered around the volume-of-interest, restricts motion artifacts, and increases the overall image quality. In line with the findings by Østergaard et al., there is no indication that STIR systematically underestimates or overestimates the severity of synovitis [10].

While there was a strong correlation between the unenhanced and enhanced synovitis subscores, there was a weak correlation for the tenosynovitis subscores. Tenosynovitis presents on MRI as fluid in the tendon sheath, thickening of the tendon sheath, and, in post-contrast sequences, as contrast enhancement of the tendon sheath [21]. As small amounts of fluid can be seen in normal tendon sheaths, tenosynovitis should be visible in at least two consecutive slices to be classified as abnormal [21], with effusions of <1.5 mm around the tendon (corresponding to grade 1, according to the updated RAMRIS) and of ≥3 mm (corresponding to the maximum grade 3) [9,21]. This classification scheme indicates that, on MRI scans, very small changes need to be detected to score tenosynovitis accurately. This could be challenging even for experienced radiologists, and potentially leads to inaccuracies in the evaluation of tenosynovitis.

In this study, the mean value for tenosynovitis-SAFE was decently lower than that for tenosynovitis-contrast. Whether this can be interpreted as higher specificity or lower sensitivity due to the lack of contrast agent is difficult to determine. Scientifically, the comparison with a gold standard would be ideal, in the sense of a histological examination, which for ethical reasons could not be performed.

Another way to investigate this question in more detail would be to compare the MRI images with a second imaging modality. Ultrasound is potentially useful for this purpose, as it plays an important role in the imaging of RA throughout the whole course of the disease [22]. MRI provides excellent soft tissue contrast and multiplanar imaging with excellent reproducibility [23]. According to the European Society of Musculoskeletal Radiology and the European League Against Rheumatism recommendations, MRI is considered the best noninvasive, observer-independent imaging modality for the evaluation of joint and tendon inflammation [24,25]. However, MRI is costly and has limited availability [22]. Advances in ultrasound transducer technology and improvements in the sensitivity of Doppler imaging have increased the usefulness of ultrasound for RA, particularly as signs of acute inflammation, such as synovial and tenosynovial effusion, are detectable [22,23]. The lack of comparison with external imaging is therefore considered a limitation in this study. Such a comparison between MRI and ultrasound is of great interest for subsequent studies.

Interestingly, the tenosynovitis subscores correlated strongly with the synovitis subscores irrespective of contrast enhancement, but not with erosion, osteitis, JSN, CRP levels, or DAS-28. Possible explanations for these findings involve the fact that synovitis and tenosynovitis tend to indicate acute inflammatory joint conditions, which renders their close association plausible. In contrast, erosion, osteitis, and JSN indicate more advanced disease stages [26]. Otherwise, the pathomechanism leading to tenosynovitis could also be largely independent of the pathomechanisms contributing to the other imaging features of RA, which would render tenosynovitis independent, also statistically.

As expected, the clinically assessed disease activity score, DAS-28, showed high and significant correlations with all other RAMRIS/RAMRIS-SAFE subscores and sum scores. CRP values were found to be significantly correlated with synovitis-contrast and DAS-28 only, which may be due to the fact that CRP is a rather nonspecific inflammatory parameter and, therefore, compared to the joint specific comprehensive assessment afforded by MRI, becomes more important in monitoring disease activity under therapy on a more global scale.

Consequently, the STIR sequence is a suitable alternative to contrast-enhanced sequences for the assessment of synovitis and tenosynovitis in the hand of RA patients. The STIR sequence is a fluid-sensitive sequence that has already been suggested in the OMERACT RAMRIS update as a possible alternative for the assessment of tenosynovitis because of this characteristic [9]. Another advantage of using the STIR sequence is that it is already included in the RAMRIS protocol for the assessment of osteitis, thus enabling time-effective measurements. Other alternative sequences might also be considered. In patients with osteoarthritis, a quantitative double-echo steady-state (qDESS) sequence has been successfully used to detect synovitis in the knee [27]. Although qDESS sequences appear to systematically underestimate the severity of synovitis [27], there is no evidence for similar observations using STIR.

Another potential future option to detect synovitis without contrast enhancement is diffusion tensor imaging (DTI). The idea emerged as altered DTI parameters were detected in brain abscesses, i.e., areas with increased occurrence of inflammatory cells [28,29]. In a pilot study on knee joints, altered DTI parameters revealed microstructural changes in the synovium, suggesting that DTI has the potential to identify synovitis [30]. Furthermore, Halston et al. used DTI to quantify the intensity of synovitis within the whole synovium [31]. However, DTI evaluation requires additional segmentation, which is currently performed manually and requires careful delineation of very thin anatomic structures, making DTI unattractive for routine clinical use.

There is concern that without exogeneous contrast enhancement, synovitis cannot be differentiated from non-inflammatory effusion. This is due to the fact that in the T1-weighted postcontrast sequences, the inflamed synovium is enhanced. Histologically proven, synovitis is also hyperintense in the T2-weighted sequences, which makes it difficult to distinguish an adjacent effusion with similar T2-hyperintense presentation from the thin synovial tissue, and thus could lead to false positive statements regarding inflammation [32,33]. As an effusion may have inflammatory as well as mechanical causes [34], there is a theoretical possibility that MRI images may mimic inflammation. However, there are no indications in our study that the omission of contrast agents leads to an overestimation of disease activity. Furthermore, the RAMRIS criteria include a combination of both increased contrast enhancement and thickening of synovial tissue to detect and classify synovitis. [4]. Thus, in high-resolution MRI imaging, theoretical uncertainties regarding contrast enhancement are mitigated by the precise assessment of synovial thickness.

Some limitations should be mentioned. First, we studied tenosynovitis-SAFE with a coronal STIR and an unenhanced T1-TSE axial sequence. For even better comparability, additional axial fluid-sensitive sequences, such as STIR or fat-saturated T2-weighted sequences, would have been desirable. Nevertheless, we assume that any tenosynovitis was detected because of the high sensitivity of the available coronal STIR sequence. Second, tenosynovitis was evaluated on the flexor tendons of the metacarpal levels only, because the tendons at the wrist level were not completely within the field of view. Third, we only evaluated one specific GBCA as administered per one particular protocol. Whether or not our findings hold true in other joints and inflammatory conditions remains to be seen. Fourth, patient sample size was limited (*n* = 31), and the results should be validated on larger cohort studies. Fifth, a heterogeneous cohort of patients was enrolled, all of whom had persistent RA but whose disease duration at the time of recruitment was variable and whose disease activity differed widely.

## 5. Conclusions

In times of persistent safety concerns regarding the use of GBCA in clinical MRI, refined imaging protocols that maintain diagnostic quality while avoiding the use of GBCA are beneficial to both patients and healthcare providers, as the degree of invasiveness and the chance of allergic reactions are reduced, patient comfort is increased, and clinical workflows and patient turnaround are further streamlined. RAMRIS-SAFE provides an imaging protocol and scoring scheme that is closely related to the well-established RAMRIS, yet without the need for contrast enhancement. Diagnostic accuracy of RA-associated destructive and inflammatory bone and joint changes is not reduced. For synovitis in particular, the STIR sequence is an accurate alternative to the post-contrast T1-weighted sequence. As patients with RA are subject to repetitive GBCA applications, non-contrast imaging protocols such as RAMRIS-SAFE should be considered.

## Figures and Tables

**Table 1:** Composition of both scores: RAMRIS-SAFE and RAMRIS.

Imaging Feature	Anatomic Location	MRI Sequence:	
		RAMRIS-SAFE (without Contrast Enhancement)	RAMRIS (with Contrast Enhancement)
Erosion	Distal radius and distal ulna	coronal T1 w/o contrast	coronal T1 w/o contrast
	All carpal bones and metacarpal bases		
	MCP joints (II-V)		
Osteitis	Distal radius and distal ulna	coronal STIR w/o contrast	coronal STIR w/o contrast
	All carpal bones and metacarpal bases MCP joints (II-V)		
Synovitis	Distal radio-ulnar joint Radiocarpal joint Intercarpal and carpometacarpal joints MCP (II-V) joints	coronal STIR, axial T1 w/o contrast	coronal and axial T1 + contrast
Tenosynovitis	Flexor tendons at the level of MCP joints	coronal STIR, axial T1 w/o contrast	coronal and axial T1 + contrast
Joint space narrowing	All radiocarpal, intercarpal, carpometacarpal, and MCP joints (*n* = 17)	coronal T1 w/o contrast	coronal T1 w/o contrast

Abbreviations are “RAMRIS” (rheumatoid arthritis MRI scoring system), “RAMRIS-SAFE” (RAMRIS Sine-Gadolinium-For-Experts), “MCP” (metacarpophalangeal), and “STIR” (Short-Tau Inversion-Recovery), “w/o” (without).

**Table 2:** Acquisition parameters of MRI sequences.

	T1 (TSE)	STIR	T1 (SE)	T1 (TSE) + Contrast	T1 (SE) + Contrast
Orientation	coronal	coronal	axial	coronal	axial
GBCA	no	no	no	yes	yes
Spectral fat suppression	no	no	yes	no	yes
TE [ms]	27	31	16	27	16
TR [ms]	862	5560	702	862	702
Slice thickness [mm]	2.5	2.5	2.5	2.5	2.5
FoV [mm^2^]	140 × 140	140 × 140	120 × 120	140 × 140	120 × 120
Inversion time [ms]	-	210	-	-	-
Matrix size [pixels^2^]	512 × 512	448 × 312	384 × 288	512 × 512	384 × 288
Voxel size [mm^3^]	0.3 × 0.3 × 2.5	0.3 × 0.3 × 2.5	0.3 × 0.3 × 2.5	0.3 × 0.3 × 2.5	0.3 × 0.3 × 2.5
Slice number [n]	17	17	20	17	20
Acquisition time [min:sec]	4:57	4:23	4:56	4:57	3:42

GBCA: Gadolinium-based contrast agent, TE: Echo time, TR: Repetition time, FoV: Field of View, TSE: Turbo Spin Echo, STIR: Short Tau Inversion Recovery, SE: Spin Echo, “-“: not applicable.

**Table 3:** Means, standard deviation (SD), and Spearman–Rho correlation matrix for imaging features of RAMRIS/RAMRIS-SAFE, DAS-28, and CRP.

	Mean	SD	1	2	3	4	5	6	7	8	9	10
1. DAS-28	4.13	1.41										
2. CRP	1.55	2.15	0.499 **									
3. Synovitis-contrast	8.39	6.27	0.469 **	0.239								
4. Synovitis-SAFE	8.52	5.59	0.463 **	0.305	0.937 **							
5. Osteitis	4.06	7.77	0.418 **	0.104	0.684 **	0.671 **						
6. Erosion	10.39	12.95	0.458 **	0.056	0.651 **	0.670 **	0.805 **					
7. JSN	8.03	12.47	0.541 **	0.208	0.564 **	0.578 **	0.712 **	0.676 **				
8. Tenosynovitis-contrast	2.68	2.57	0.023 **	−0.068	0.579 **	0.515 **	0.196 **	0.279 **	0.130 **			
9. Tenosynovitis-SAFE	2.26	2.42	0.085 **	0.052	0.390 **	0.381 **	0.304 **	0.229 *	0.212 **	0.380 **		
10. RAMRIS	33.55	34.94	0.480 **	0.131	0.867 **	0.846 **	0.852 **	0.854 **	0.787 **	0.481 **	0.374 *	
11. RAMRIS-SAFE	33.26	34.36	0.491 **	0.166	0.839 **	0.859 **	0.863 **	0.873 **	0.812 **	0.378 **	0.401 *	0.976 **

DAS-28: Disease Activity Score 28, CRP: C-reactive protein, SAFE: Sine-Gadolinium-For-Experts, JSN: joint space narrowing, RAMRIS: Rheumatoid Arthritis Magnetic Resonance Imaging Score. **. *p* ≤ 0.01, *. *p* ≤ 0.05.

## Data Availability

Data can be provided by the authors upon reasonable request.

## References

[1] Strand V., Singh J.A. (2007). Improved health-related quality of life with effective disease-modifying antirheumatic drugs: Evidence from randomized controlled trials. Am. J. Manag. Care.

[2] Wilson R.L. (1986). Rheumatoid arthritis of the hand. Orthop. Clin. N. Am..

[3] Zochling J., Braun J. (2009). Mortality in rheumatoid arthritis and ankylosing spondylitis. Clin. Exp. Rheumatol..

[4] Singh J.A., Saag K.G., Bridges S.L., Akl E.A., Bannuru R.R., Sullivan M.C., Vaysbrot E., McNaughton C., Osani M., Shmerling R.H. (2016). 2015 American College of Rheumatology Guideline for the Treatment of Rheumatoid Arthritis. Arthritis Rheumatol..

[5] Sewerin P., Schleich C., Vordenbäumen S., Ostendorf B. (2018). Update on imaging in rheumatic diseases: Cartilage. Clin. Exp. Rheumatol..

[6] Baraliakos X., Braun J., Conaghan P.G., Østergaard M., Pincus T. (2018). Update on imaging in rheumatic diseases. Clin. Exp. Rheumatol..

[7] Frenken M., Schleich C., Brinks R., Abrar D.B., Goertz C., Schneider M., Ostendorf B., Sewerin P. (2019). The value of the simplified RAMRIS-5 in early RA patients under methotrexate therapy using high-field MRI. Arthritis Res. Ther..

[8] Conaghan P.G., Østergaard M., Troum O., Bowes M.A., Guillard G., Wilkinson B., Xie Z., Andrews J., Stein A., Chapman D. (2019). Very early MRI responses to therapy as a predictor of later radiographic progression in early rheumatoid arthritis. Arthritis Res. Ther..

[9] Østergaard M., Peterfy C.G., Bird P., Gandjbakhch F., Glinatsi D., Eshed I., Haavardsholm E.A., Lillegraven S., Bøyesen P., Ejbjerg B. (2017). The OMERACT Rheumatoid Arthritis Magnetic Resonance Imaging (MRI) Scoring System: Updated Recommendations by the OMERACT MRI in Arthritis Working Group. J. Rheumatol..

[10] Østergaard M., Conaghan P.G., O’Connor P., Szkudlarek M., Klarlund M., Emery P., Peterfy C., Genant H., McQUEEN F.M., Bird P. (2009). Reducing Invasiveness, Duration, and Cost of Magnetic Resonance Imaging in Rheumatoid Arthritis by Omitting Intravenous Contrast Injection—Does It Change the Assessment of Inflammatory and Destructive Joint Changes by the OMERACT RAMRIS?. J. Rheumatol..

[11] Olchowy C., Cebulski K., Łasecki M., Chaber R., Olchowy A., Kałwak K., Zaleska-Dorobisz U. (2017). The presence of the gadolinium-based contrast agent depositions in the brain and symptoms of gadolinium neurotoxicity—A systematic review. PLoS ONE.

[12] Tedeschi E., Caranci F., Giordano F., Angelini V., Cocozza S., Brunetti A. (2017). Gadolinium retention in the body: What we know and what we can do. Radiol. Med..

[13] Quattrocchi C.C., Van Der Molen A.J. (2017). Gadolinium Retention in the Body and Brain: Is It Time for an International Joint Research Effort?. Radiology.

[14] Stojanov D., Aracki-Trenkic A., Benedeto-Stojanov D. (2016). Gadolinium deposition within the dentate nucleus and globus pallidus after repeated administrations of gadolinium-based contrast agents—Current status. Neuroradiology.

[15] Cowling T., Frey N. (2019). Macrocyclic and Linear Gadolinium Based Contrast Agents for Adults Undergoing Magnetic Resonance Imaging: A Review of Safety.

[16] Dekkers I.A., Roos R., Van Der Molen A.J. (2018). Gadolinium retention after administration of contrast agents based on linear chelators and the recommendations of the European Medicines Agency. Eur. Radiol..

[17] Aletaha D., Neogi T., Silman A.J., Funovits J., Felson D.T., Bingham C.O., Birnbaum N.S., Burmester G.R., Bykerk V.P., Cohen M.D. (2010). 2010 Rheumatoid arthritis classification criteria: An American College of Rheumatology/European League Against Rheumatism collaborative initiative. Ann. Rheum. Dis..

[18] Van Riel P.L., Renskers L. (2016). The Disease Activity Score (DAS) and the Disease Activity Score using 28 joint counts (DAS28) in the management of rheumatoid arthritis. Clin. Exp. Rheumatol..

[19] Cohen S., Kamarck T., Mermelstein R. (1983). A global measure of perceived stress. J. Health Soc. Behav..

[20] Landis J.R., Koch G.G. (1977). The Measurement of Observer Agreement for Categorical Data. Biometrics.

[21] Haavardsholm E.A., Østergaard M., Ejbjerg B.J., Kvan N.P., Kvien T.K. (2007). Introduction of a novel magnetic resonance imaging tenosynovitis score for rheumatoid arthritis: Reliability in a multireader longitudinal study. Ann. Rheum. Dis..

[22] Di Matteo A., Mankia K., Azukizawa M., Wakefield R.J. (2020). The Role of Musculoskeletal Ultrasound in the Rheumatoid Arthritis Continuum. Curr. Rheumatol. Rep..

[23] Docking S.I., Ooi C.C., Connell D. (2015). Tendinopathy: Is Imaging Telling Us the Entire Story?. J. Orthop. Sports Phys. Ther..

[24] Sudoł-Szopińska I., Jans L., Teh J. (2017). Rheumatoid arthritis: What do MRI and ultrasound show. J. Ultrason..

[25] Sudoł-Szopińska I., Jurik A.G., Eshed I., Lennart J., Grainger A., Østergaard M., Klauser A., Cotten A., Wick M.C., Maas M. (2015). Recommendations of the ESSR Arthritis Subcommittee for the Use of Magnetic Resonance Imaging in Musculoskeletal Rheumatic Diseases. Semin. Musculoskelet. Radiol..

[26] McQueen F.M. (2009). The MRI view of synovitis and tenosynovitis in inflammatory arthritis: Implications for diagnosis and management. Ann. N. Y. Acad. Sci..

[27] De Vries B.A., Breda S.J., Sveinsson B., McWalter E.J., Meuffels D.E., Krestin G.P., Hargreaves B.A., Gold G.E., Oei E.H.G. (2021). Detection of knee synovitis using non-contrast-enhanced qDESS compared with contrast-enhanced MRI. Arthritis Res. Ther..

[28] Gupta R.K., Nath K., Prasad A., Prasad K., Husain M., Rathore R., Husain N., Srivastava C., Khetan P., Trivedi R. (2008). In Vivo Demonstration of Neuroinflammatory Molecule Expression in Brain Abscess with Diffusion Tensor Imaging. Am. J. Neuroradiol..

[29] Gupta R.K., Hasan K.M., Mishra A.M., Jha D., Husain M., Prasad K.N., Narayana P.A. (2005). High Fractional Anisotropy in Brain Abscesses versus Other Cystic Intracranial Lesions. Am. J. Neuroradiol..

[30] Agarwal V., Kumar M., Singh J.K., Rathore R.K.S., Misra R., Gupta R.K. (2009). Diffusion tensor anisotropy magnetic resonance imaging: A new tool to assess synovial inflammation. Rheumatology.

[31] Sandford H.J.C., MacKay J.W., Watkins L.E., Gold G.E., Kogan F., Mazzoli V. (2021). Gadolinium-free assessment of synovitis using diffusion tensor imaging. NMR Biomed..

[32] König H., Sieper J., Wolf K.J. (1990). Rheumatoid arthritis: Evaluation of hypervascular and fibrous pannus with dynamic MR imaging enhanced with Gd-DTPA. Radiology.

[33] Stomp W., Krabben A., Van Der Heijde D., Huizinga T.W.J., Bloem J.L., Østergaard M., van der Helm-van Mil A.H., Reijnierse M. (2015). Aiming for a simpler early arthritis MRI protocol: Can Gd contrast administration be eliminated?. Eur. Radiol..

[34] Damman W., Liu R., Reijnierse M., Rosendaal F.R., Bloem J.L., Kloppenburg M. (2021). Effusion attenuates the effect of synovitis on radiographic progression in patients with hand osteoarthritis: A longitudinal magnetic resonance imaging study. Clin. Rheumatol..

